# Research progress on the mechanism of interleukin-1β on epiphyseal plate chondrocytes

**DOI:** 10.1186/s40001-022-00893-8

**Published:** 2022-12-27

**Authors:** Ziyuan Tong, Xu Yang, Jianjun Li

**Affiliations:** 1 Department of Orthopedics, Shengjing Hospital of China Medical University, Shenyang, 114000 Liaoning China; 2 Department of Anesthesiology, Shengjing Hospital of China Medical University, Shenyang, 114000 Liaoning China

**Keywords:** Interleukin-1β (IL-1β), Epiphyseal chondrocytes, Bone bridge, Collagen type II, Oxidative stress

## Abstract

Epiphyseal plate injury, a common problem in pediatric orthopedics, may result in poor bone repair or growth defects. Epiphyseal plate, also known as growth plate is a layer of hyaline cartilage tissue between the epiphysis and metaphyseal and has the ability to grow longitudinally. Under normal physiological conditions, the epiphyseal plate has a certain axial resistance to stress, but it is fragile in growth phase and can be damaged by excessive stress, leading to detachment or avulsion of the epiphysis, resulting in life-long devastating consequences for patients. There is an obvious inflammatory response in the phase of growth plate injury, the limited physiological inflammatory response locally favors tissue repair and the organism, but uncontrolled chronic inflammation always leads to tissue destruction and disease progression. Interleukin-1β (IL-1β), as representative inflammatory factors, not only affect the inflammatory phase response to bone and soft tissue injury, but have a potentially important role in the later repair phase, though the exact mechanism is not fully understood. At present, epiphyseal plate injuries are mainly treated by corrective and reconstructive surgery, which is highly invasive with limited effectiveness, thus new therapeutic approaches are urgently needed, so a deeper understanding and exploration of the pathological mechanisms of epiphyseal plate injuries at the cellular molecular level is an entry point. In this review, we fully introduced the key role of IL-1 in the progression of epiphyseal plate injury and repair, deeply explored the mechanism of IL-1 on the molecular transcript level and endocrine metabolism of chondrocytes from multiple aspects, and summarized other possible mechanisms to provide theoretical basis for the clinical treatment and in-depth study of epiphyseal plate injury in children.

## Introduction

The growth plate assumes responsibility for children’s longitudinal growth of long bones. However, as the weakest part of long bone, it is vulnerable to damage and have limited regenerative capacity, which may often fail to be fully repaired after injury, resulting in some patients being severely affected for the later growth failure. Inhibition of the growth function of the epiphyseal plate generally occurs for two reasons: (1) due to the cartilage damage or blood supply disorder, the ability of the epiphyseal plate growth zone is reduced, leading to premature closure; (2) special types of fracture like Salter–Harris type III and IV epiphyseal plate fractures misaligned healing and local formation of bone bridges, resulting in restrained growth. Incomplete closure or even premature arthrogryposis may result from complete growth failure, differences in limb lengths, and angulation deformities, a series of complications that can significantly affect growth and development in children. It is therefore necessary to properly assess and manage growth plate injuries. The procedure of fracture healing involves complex processes such as inflammation inflammatory reaction, endochondral ossification and bone reconstruction [1, 2]. The immune response at the injured site is of critical importance to affect fracture healing, which rapidly forms hematoma, chemotactic and recruit a large number of inflammatory cells [3], releasing the cytokine IL-1β, TNF-α, etc. It can be metabolized physiologically when pro-inflammatory and anti-inflammatory cytokines work together to maintain homeostasis. However, due to the limited regeneration and self-repair ability of this site, without intervention, lots of inflammatory factors infiltrate like IL-1β, causing dynamic imbalance of pro-inflammatory and anti-inflammatory cytokines, triggering chondrocyte apoptosis and metabolic abnormalities resulting in bone growth defects of the involved limb [4, 5]. It is consistent with the research using growth plate injured rats by Fiona H. Zhou et al., which showed that IL-1β may play an important role in early acute inflammatory events and later bone bridge formation and remodeling [6]. However, there are few research about the detailed mechanism of IL-1β on epiphyseal plate chondrocytes at present. With the continuous development of studies on cartilage-derived diseases such as traumatic epiphyseal plate closure and osteoarthritis, increasing attention is being gained to IL-1β action. Therefore, this article focuses on clarifying the mechanism of action of IL-1β on the effect on epiphyseal plate chondrocytes, provides new perspectives and ideas to furnish a theoretical basis for clinical applications.

## Biological function of IL-1

The IL-1 family concludes 11 members, and these cytokines have the same c-terminal three-dimensional structure. In 1984, IL-1 cDNA isolated from human body first proved that IL-1 has two different biochemical forms, namely IL-1α and IL-1β [7]. IL-1β is mainly secreted from macrophages, with a molecular weight of about 17 kDa and widely present in various tissues and organs in human body. IL-1β is an inflammatory cytokine with the same receptor complex as IL-1α and acts on the same cytokine receptor for signal regulation, which can trigger inflammation, especially when tissues are damaged. But the effects also differ due to differences in how they are produced, where IL-1β is mainly synthesized by phagocytes [8]. The pathogenic effect of IL-1β has been fully verified in auto-inflammatory diseases, mainly on account of gain-of-function mutations in genes encoding mature inflammasome. Studies have also confirmed that IL-1β is locally induced in the bone marrow niche in response to injury, which contributes to bone marrow emergency generation [9, 10]. Autoimmune diseases, diabetes mellitus, gout, and neurodegenerative diseases, among others, also have its involvement [8, 11, 12]. As the best-characterized member of this family [8], the secretion of IL-1β is tightly regulated, requiring inflammatory activation as a second stimulus. Inflammatory activation makes maturation of inflammatory caspases, followed by cleavage of the pro-IL-1β into active forms, and secret subsequently [13, 14]. In addition, due to the polymorphism of IL-1β, its function is not only limited to the body inflammatory response, but is associated with multiple system pathological functions [15–17]. In 1989, Yamashita et al. confirmed that IL-1β can be produced by mature chondrocytes by immunohistochemical localization of IL-1 in human epiphyseal plate and cartilage canal tissue [18]. Normal joint synovial fluid contains trace amount of IL-1β. It can also be observed in cultured chondrocytes and synovial cells [19]. It indicates that IL-1β derived from chondrocytes may have an important impact in hypertrophy, cartilage revascularization and bone formation. Jenei-Lanzl Z et al. described a link between IL-1β and bone disorders in different subpopulations [20], IL-1β has effect on chondrocytes through catabolism, a process that involves upregulation of polymerases and matrix metalloproteinase (MMPs) and is further self-upregulated in chondrocytes through a positive feedback mechanism. In addition, a certain degree of mechanical strain can also induce IL-1β expression in chondrocytes in osteoarthritis. The function of IL-1β is far more than these, and it was the availability of its correlative studies with chondrocytes that gave us a new entry point/direction into the study of poor healing after epiphyseal plate injury.

## How IL-1β affects the chondrocytes

### Degradation of the cartilage extracellular matrix

#### Downregulation of IL-1β on SOX-9 expression

Earlier studies by M et al. found that IL-1β inhibited mRNA expression of proteoglycan and collagen type II, and significantly prevented synthesis of proteoglycan, resulting in ATDC5 chondrocyte dynamics and metatarsal growth restriction [21]. Collagen type II, one of the principal extracellular matrix components abundant in cartilage, combines with proteoglycan to maintain the cellular structure acting as a skeleton for chondrocyte attachment and migration, and interacts with chondrocytes to effectively maintain the chondrogenic phenotype, While the decrease of their synthesis and expression will lower the potential for recovery results in loss of the chondrocyte phenotype. As a key transcription factor in chondrocytes, sox-9 plays a key role in the process of cartilage development, activating the transcription of many cartilage specific genes, such as collagen-2 (COL2A1) and cartilage oligomeric protein (COMP), which encode the extracellular matrix (ECM) component, and directly regulating Col2a1 transcription process, especially by targeting specific binding sites located in introns [22, 23]. Specific deletion of SOX9 leads to severe cartilage dysplasia in mice before and after mesenchymal condensation [24]. This was further supported by the fact that SOX9 transcription levels were significantly reduced in osteoarthritis with degradation of extracellular matrix, while when transduced to overexpress in human chondrocytes, it significantly stimulated the synthesis of proteoglycan and collagen II to restore extracellular matrix and promote the re-expression of cartilage phenotype [25, 26]. In addition, silencing of SOX9 reversed the protective effect of etomidate on ECM degradation components in an in vitro injury model of chondrocytes stimulated by glycation end-products (AGEs) [27]. Machiyuan et al. observed for the first time that degradation of type II collagen and SOX-9 was only regulated by of nuclear receptor subfamily 4A group a member 3 (NR4A3). Further studies showed that IL-induced changes in NR4A3 in chondrocytes affected cartilage matrix degradation [28, 29]. What’s more, the expression of collagen II and SOX-9 decreased in chondrocytes under IL-1β intervention, while F-box/WD repeat-containing protein 7 (FBW7) promoted the integral role of collagen type II, proteoglycan and Sox-9 in cartilage to correct chondrocytic disorders [30]. Xu Z et al. found that the activation of G protein coupled receptor 120 (GPR120) after exposure to IL-1β in ATDC5 chondrocytes can reverse the expression of collagen type II and proteoglycan via blocking the downregulation of Sox-9, to reduce the inflammation induced by IL-1β [31]. The above studies illustrate that the elevation of IL-1β content can negatively regulate SOX-9 after injury occurs, which in turn leads to inhibition of proteoglycan and collagen type II synthesis, finally causing cartilage extracellular matrix degradation.

#### Induction of IL-1β on MMP synthesis

Sox-9 expression in chondrocyte in response to IL-1β was reduced, as was the matrix metalloproteinase family (MMPs). MMPs, a super family of protease widely found in connective tissues, whose activity is regulated by zinc ions, are mainly responsible for extracellular matrix degradation and tissue remodeling. Luteolin has been proven to exert anti-inflammatory properties, and Junliang Fei et al., by evaluating the expression of various indicators in chondrocytes that were intervened with IL-1β for 24 h, suggested that luteolin significantly decreased MMP9 and MMP13 synthesis which promoted by IL-1β, effectively reversed collagen type II degradation [32]. Bone morphogenetic protein 2 (BMP2) is a known indicator of osteogenesis. IL-1β stimulates chondrocytes and increases the expression levels of BMP2 and MMP13 by targeting the MEK/ERK/SP1 pathway, enhancing cartilage structural remodeling and cartilage degradation, which leads to chondrocyte degeneration [33]. In the investigation of osteogenic differentiation process luteolin was found that, in periodontal ligament cells, on the one hand, was able to dose-dependently increase BMP2 expression to promote osteogenic differentiation, on the other hand, was to simultaneously antagonize the negative effect of IL-1β-promoted MMP production on cartilage production [34, 35]. In fibrocartilage-derived cells of the temporomandibular joint (TMJ), IL-1β increased the fibro-chondrocyte proteoglycan ADAMTS4 and ADAMTS5 expression, as well as strongly increased MMP-13 expression, then inducing cartilage damage [36]. In addition, disruption of collagen II and proteoglycan is an essential feature of cartilage in patients with intervertebral disc degeneration (IDD). It is found that reduction of cartilage-derived morphogenetic protein-1 (CDMP-1) was dose-dependent, while appropriate supplementation of CDMP-1 contributed to collagen II and proteoglycan synthesis and inhibited MMP-9 and MMP-13 breakdown after using IL-1β to intervene in nucleus pulposus cells [37]. Also, Wei Qi et al. treated human nucleus pulposus cells (HNPC) derived from the notochord under IL-1β intervention with tyrosol, a multi-component compound with anti-inflammatory properties, and found that tyrosol inhibited IL-1 through SIRT1/PI3K/AKT pathway to reverse the upregulation of MMP-3, MMP-9 and MMP-13, which can just reduce the degradation of type II collagen in chondrocytes [38]. In addition, Elsa Mével et al. study showed that hydroxytyrosol (HT), an olive major extract, exerted anti-osteoarthritis effects in a post-traumatic animal model and exhibited anti-inflammatory and chondroprotective effects in IL-1β-stimulated primary cultured rabbit chondrocytes [39]. Exploring the effects of tyrosol and similar compounds in chondrocyte culture in vitro or in an epiphyseal plate injury model in vivo would hopefully represent a breakthrough point for the treatment of this condition in the clinic. On the whole, IL-1β ultimately leads to cellular abnormalities by increasing the synthesis of MMPs, causing decomposition in collagen type II and thereby degrading cartilage extracellular matrix.

#### Effect of IL-1β at gene level

Accumulating evidence so far proves that RNA plays important roles in various diseases. Numerous studies have shown that IL-1β autophagy in cartilage was stimulated at the initial stage of inflammation, but was eventually significantly inhibited. It was found that upregulating ciRS-7 abnormally expressed in OA could enhance IL-cartilage degradation induced by IL-1β [40]. Beyond that, circRNA.33186 was frequently upregulated in chondrocytes treated with IL-1β, by knocking down circRNA.33816, MMP-13 was found decreased in chondrocytes by Zhou et al., while collagen type II increased, which accelerated proliferation of chondrocytes but simultaneously inhibited apoptosis [41]. circRNA.33186 is circular and mainly exists in the cytoplasm. Early studies found that circular RNA can competitively bind to miRNA [42]. Some scholars screened that there was an obviously negative interaction between miR-127-5P and circRNA.33186 through luciferase, which targeted MMP13 to regulate the catabolic function of IL-1β-treated chondrocytes, confirming that circRNA.33186 could directly or indirectly affect MMP13 through miR-127-5P, resulting in abnormal chondrocyte function [43]. At present, it has been recognized that miRNA and lncRNA are indispensable in the occurrence and development of diseases. They are of great importance to life activities, such as cell cycle regulation and cellular differentiation. Concretely, LncRNA MALAT1 was shown to be upgraded in IL-1β-treated chondrocytes. Ying Zhang et al. demonstrated that in the same cell model, MALAT1 targets miR-150-5p to regulate Akt3 indirectly, and competitively binds with miR-150-5p to inhibit proteoglycan and collagen II expression, reduce cell proliferation as well [44]. Moreover, MALAT1 directly binds to MiR-145, which is negatively regulated. Overexpression of MALAT1 suppressed chondrocyte viability after IL-1β promotion and degraded extrachondral matrix, which was opposite to the effect results after miR-145 upregulation [45]. Besides, MALAT1 has been shown to regulate chondrocytes through the regulation of miR-515-3P or miR-181a-5p [46, 47]. There is another RNA worth mentioning called LncRNA snhg5, which was downregulated in OA and targeted to regulate H_3_ histone family 3B (H3F3B) expression through miR-10a-5p to enhance apoptosis caused by IL-1β [48]. It is also interesting to note that both SNHG5 and MALAT1 could protect stimulated chondrocytes with IL-1β by regulating miR-181a-5p, but the two target proteins were different [49]. In addition, biochemical analysis predicted high mobility group box 1 (HMGB1) was a target agent of miR-140-5p, and its overexpression reduced HMGB1, thereby suppressing inflammatory responses and apoptosis in IL-1β-treated chondrocytes [50]. In a study by Jing Wang et al., increased miR-98 expression was found in chondrocytes under IL-1β intervention, while inhibition of miR-98 effectively reduced cell apoptosis, suggesting that IL-1β regulated chondrocyte apoptosis-related proteins through mir-98 [51]. The above studies proved that IL-1β could affect chondrocyte matrix catabolism by affecting different proteins of interest with RNA targeting, and in the future, RNA regulation could be used as an entry point to provide a theoretical reference for clinical prevention and curing abnormal changes in chondrocytes.

### Facilitation of adipogenesis

Among patients with orthopedic diseases, obese individuals deserve our attention for numerous studies have found that mediators of joint degeneration are derived from adipose paracrine signaling [52–54]. IL-1β plays a vital role on lipid metabolism via regulating lipase activity and negatively adjusts cartilage, there are reports of an association between IL-1β and obesity independent of population [55, 56]. In obese humans, adipocytokines, like leptin and adiponectin secretion by adipocytes is increased [57–60], which regulates inflammatory response, cartilage catabolic activity and bone remodeling, and is involved in the occurrence and development of obesity-induced osteoarthritis [61–64]. Leptin, an adipose tissue-derived adipokine with multiple immune and physiological functions [65], suppressing eating and increasing thermogenesis, and participates in multiple immune inflammatory responses [66]. T Simopoulou et al. demonstrated that leptin and its receptors were outstandingly enhanced at progressive stages, and the expression of leptin mRNA is closely related to BMI, moreover, IL-1β, MMP-9 and MMP-13 protein were also increased, with adverse effects on chondrocytes [67]. Adiponectin is another factor secreted by adipose tissue. Early studies established that adiponectin reduces cartilage extracellular matrix degradation and cartilage destruction resulting from increased MMP-13 induced by IL-1β [68]. In 2007, Simons P j et al. also found that adiponectin secretion was significantly downregulated by chronic exposure of adipocytes to IL-1β [69]. Later, T Delessa Challa et al. found that adiponectin (0.5 ug/ml) could promote mouse ATDC5 chondrocyte cell line proliferation and elevated the expression of collagen II, proteoglycan, Runx2, etc., demonstrating that low-level adiponectin effected positively on chondrocyte proliferation and differentiation [70]. It follows that leptin secretion is increased in obesity, bringing about pro-inflammatory effects, whereas adiponectin synthesis, which is anti-inflammatory, is decreased. While an in-depth study of its specific mechanism may be able to provide a new direction for us in the clinic for the treatment of obese patients with epiphyseal plate fractures as well as the prevention of complications.

What’s more, adipose tissue, made up of developing and mature adipocytes as well as a wide range of immune cells [71], though of a low-grade nature, is sufficient to cause negative effects on distant organ function [72]. Initially, obesity-related comorbidities were thought to dominate in diabetes mellitus type 2 (T2DM) due to growth of the global economy [73]. Obesity may lead to disturbed homeostasis between adipocytes and immune cells, and cause M1 macrophages fragmentation and polarization, activating the NLRP3 inflammatory to massively secrete IL-1β to exacerbate pro-inflammatory responses, a process that has been found to be involved in type 1 diabetes mellitus (T1DM) [74–76]. High levels of IL-1β, positively correlated with the severity of diabetes, confer insulin resistance in obese individuals [77, 78]. Evidence suggests that prolonged elevation of IL-1β promoted insulin expression, stimulated glucose uptake and aggravated macrophage inflammation, resulting in severe pathological metabolic processes [79]. In a study of the relationship between T2MD, obesity and skeleton, Francesca Vigevano et al. collected and analyzed data from 112 obese men and found that group with T2DM and obesity had more bone disease than those without T2DM [80]. This may indicate that high levels of IL in obesity-induced chronic inflammation leads to adverse skeletal reactions.

Since the twenty-first century, the problem of adolescent obesity has become increasingly severe, as a matter of fact, low-grade chronic inflammation leads to insulin resistance and diabetes, including type I diabetes mellitus(T1DM) [81]. Abnormal fat metabolism in obese children has become an early manifestation of diabetes, T1DM, also called insulin-dependent diabetes. Clinical statistics found that T1DM accounted for 5–10% of all diabetes cases and has a predilection for children or adolescents [82]. In general, T1DM is considered an immune disease [83], and the role of NLRP3 in T1DM remains to be investigated; whereas, recent studies point to a greater incidence of T1DM with obesity [73], possibly for that obesity leads to immunogenicity and glucose dysregulation, thereby contributing to insulin resistance in patients with T1DM. In addition, toll like receptors (TLRs), one pattern recognition molecule, which may be a biomarker in the early stage of T1DM, induce the production of IL-1β [84]. So further understanding of the role of IL-1β in T1DM may improve prognosis of fracture patients with juvenile T1DM. Earlier studies detected significantly higher IL-1β levels and lower insulin levels in patients with T1DM [84, 85] which correlated with IL-1β inducing pro-inflammatory factors migration to pancreatic islets and exerting cytotoxic effects [86], IL-1β reduced insulin-induced glucose transport in adipocytes, leading to lipid accumulation in muscle versus liver and deleterious effects. Studies have shown that skeletal and muscle health was affected by poorly controlled T1DM disease course in children [87].

It can be seen that obesity may cause an inflammatory response with elevated IL-1β levels, increasing the probability of developing diabetes while improving the risk of fracture and affecting fracture healing. Once the epiphyseal plate is injured, more IL-1β infiltration will increase the possibility of poor prognosis. In general, IL-1β is closely related to lipid and cartilage, especially in the context of the general increase of obese children, thus continued exploration of the relationship between adipocytes and chondrocytes and deeper investigation of obesity and bone diseases may in the future clinically provide a new direction for treating bone fractures in obese people, especially adolescents.

### Promotion of oxidative stress

The chief reason for oxidative stress is disequilibrium between the generation of intracellular reactive oxygen species (ROS) and the scavenging effects of antioxidant ability, which is one main cause of chronic inflammation. In OA, oxidative stress has a certain influence on chondrocytes. Cellular ROS originates from mitochondrial respiratory chain [88], mainly produced by reduced NADPH oxidases (NOX) in chondrocytes [89], which usually exists in cells at a low level and is vital to maintain cell function and stability [90]. Previous studies have found that ROS oxidative stress make IL-1β and other inflammatory mediators highly up-regulated [91–93], which can induce the production of ROS and MMPs to degrade extracellular matrix [94, 95]. As a key link of classical inflammatory pathway, NF-κB participates in the regulation of a variety of genes, and it is a necessary molecule involved in the pathophysiological changes of cartilage [96].

Chondrocyte with hyperoside (Hyp) preconditioning saved ROS overproduction and chondrocyte apoptosis induced by IL-1β, playing an anti-inflammatory role by partially inhibiting NF-κB signaling pathway [97], so as simvastatin[98] and theobromine [99]. Garlic derived S-Allyl mercaptocysteine (SAMC) also has cartilage protection, but it mainly activates nuclear factor-E2 related factor (Nrf2), accompanied by downregulation of NOX4, resulting in improvement of collagen damage and maintenance of redox homeostasis [100]. At present, it is known that transcription factors that maintain cell redox balance and signal transduction can reduce intracellular oxidative stress damage [101]. Yao x et al. observed that Ferrostatin-1, a specific inhibitor of iron death, can reduce IL-1β-induced ROS accumulation, activate Nrf2 antioxidant system and rescue the expression of type II collagen [102]. Similarly, studies have found that a natural naphthoquinone compound β-hydroxyisovalerylshikonin (β-HIVs) can also inhibit IL-induced ROS production and chondrocyte metabolism through Nrf2, and downregulate the expression of ADAMTS5 and MMP13 [103]. Activation of nuclear receptor subfamily 1 group D member 1 (NR1D1) enhance Nrf2 pathway as well [104]. What’s more, Recent studies have shown that Licochalcone a (Lico a) can reduce the level of IL-1β and NLRP3 in vitro, and inhibit cell death via Nrf2/HO-1/NF-κB signal axis, to improve the degradation of cartilage extracellular matrix [105]. Normoline (NOM)-pretreated chondrocytes were also proved to inhibit NF-κB signal transduction by dissociating kelch like ECH associated protein 1 (Keap1) /Nrf2 path, effectively inhibiting inflammatory factor recruitment and ROS over regulation [106]. In conclusion, the weakening of NF-κB pathway and the activation of Nrf2 pathway can reduce IL-1β and ROS, reducing the degree of oxidative stress and protect chondrocytes.

It is worth mentioning, NOX4 is the only subtype expressed in chondrocytes in the NOX family, mainly cause ROS overproduction in chondrocytes after IL-1β stimulation [107, 108]. Besides, Heme oxygenase-1 (HO-1) is very important for NOX4 activity, which significantly downregulates the expression of MMP-1and NOX4 in IL-1β-treated chondrocytes [109]. Later studies further confirmed that IL-1β mediated NOX4 to stimulate the upregulation of MMP-1 and MMP-13 [110], which is consistent with the research results of Fu D et al. [111]. The regulation of oxidative stress and the relief of neuroinflammation may provide an effective reference for clinical treatment.

## Conclusion

The repair of epiphyseal cartilage injury is regulated by many factors, and the inflammatory response is directly related to the growth, development and structural function of the repaired bone. Studies have shown that IL-1β levels are significantly elevated which can affect chondrocytes through a variety of ways, including the reduction of SOX-9 expression, the promotion of MMP synthesis, and further inhibit the interaction between proteoglycan and collagen II to achieve EMC degradation. In addition, IL-1β acts directly or indirectly on chondrocytes through lipids to promote lipolysis, resulting in abnormal levels of adiponectin and leptin, affecting lipid metabolism, and promoting the development of inflammation. In addition, IL-1β also causes irreversible damage to chondrocytes through multiple miRNA and circRNA targeting effects or enhancement of the response to oxidative stress, which has been effectively demonstrated in a variety of cellular or animal experiments and represents some of the advance in the understanding of the mechanisms of osteoarticular chondrocytes and growth plates repair. Further experimental work is also needed to deeply investigate the exact mechanism by which IL-1β affects growth plate chondrocytes to reduce or eliminate the effects of various harmful factors on the epiphyseal plate as early as possible to give new research directions in repair after epiphyseal plate injury.

## Data Availability

Not applicable.
